# Phenotype of postural instability/gait difficulty in Parkinson disease: relevance to cognitive impairment and mechanism relating pathological proteins and neurotransmitters

**DOI:** 10.1038/srep44872

**Published:** 2017-03-23

**Authors:** Li-Jun Zuo, Ying-Shan Piao, Li-Xia Li, Shu-Yang Yu, Peng Guo, Yang Hu, Teng-Hong Lian, Rui-Dan Wang, Qiu-Jin Yu, Zhao Jin, Ya-Jie Wang, Xiao-Min Wang, Piu Chan, Sheng-Di Chen, Yong-Jun Wang, Wei Zhang

**Affiliations:** 1 Department of Neurology, Beijing Tiantan Hospital, Capital Medical University, Beijing, 100050, China; 2 Department of Geriatrics, Beijing Tiantan Hospital, Capital Medical University, Beijing, 100050, China; 3 Core Laboratory for Clinical Medical Research, Beijing Tiantan Hospital, Capital Medical University, Beijing, 100050, China; 4 Department of Neurobiology and Physiology, Capital Medical University, Beijing, 100069, China; 5 Key Laboratory for Neurodegenerative Disorders of the Ministry of Education, Capital Medical University, Beijing, 100069, China; 6 Beijing Institute for Brain Disorders, Capital Medical University, Beijing, 100069, China; 7 Beijing Key Laboratory on Parkinson disease, Beijing, 100053, China; 8 Department of Neurobiology, Beijing Institute of Geriatrics, Xuanwu Hospital of Capital Medical University, Beijing, 100053, China; 9 Department of Neurology, Ruijin Hospital Affiliated to Shanghai Jiaotong University School of Medicine, Shanghai, 200025, China; 10 China National Clinical Research Center for Neurological Diseases, Beijing, 100050, China

## Abstract

Parkinson disease (PD) is identified as tremor-dominant (TD) and postural instability and gait difficulty (PIGD) phenotypes. The relationships between motor phenotypes and cognitive impairment and the underlying mechanisms relating pathological proteins and neurotransmitters in cerebrospinal fluid (CSF) are unknown. We evaluated the motor symptoms and cognitive function by scales, and detected the levels of pathological proteins and neurotransmitters in CSF. TD group and PIGD group had significantly higher levels of total tau, tau phosphorylated at the position of threonine 181(P-tau181t), threonine 231, serine 396, serine 199 and lower β amyloid (Aβ)_1–42_ level in CSF than those in control group; PIGD group had significantly higher P-tau181t level and lower Aβ_1–42_ level than those in TD group. In PD group, PIGD severity was negatively correlated with MoCA score and Aβ_1–42_ level in CSF, and positively correlated with Hoehn-Yahr stage and P-tau181t level in CSF. In PIGD group, PIGD severity was negatively correlated with homovanillic acid (HVA) level in CSF, and HVA level was positively correlated with Aβ_1–42_ level in CSF. PIGD was significantly correlated with cognitive impairment, which underlying mechanism might be involved in Aβ_1–42_ aggregation in brain and relevant neurochemical disturbance featured by the depletion of HVA in CSF.

Parkinson disease (PD) is a common and progressively neurodegenerative disorder with motor symptoms and a variety of non-motor symptoms. Cognitive impairment of PD has been paid more attention to in recent years. However, motor symptoms and cognitive deficits in PD are heterogeneous in age of onset^1^, clinical manifestations^2^ and disease progression^3^. PD patients are mainly divided into two phenotypes based on motor symptoms: tremor-dominant (TD) and postural instability gait difficulty (PIGD) phenotypes^4^. Previous studies showed that PIGD phenotype or change from TD to PIGD phenotype served as a higher risk of cognitive decline and the development of dementia^5^ in later stage of PD^6^. However, the above studies were small size, and focused on the clinical manifestations. The current study explored the relationship between different motor phenotypes and cognitive function in PD patients and the underlying mechanisms. Since PD is an α-synucleinopathy, α-synuclein is associated with motor symptom^7^,^8^ and several non-motor symptoms, such as cognitive impairment^9^, fatigue^10^, apathy^11^ and rapid eyes movement (REM) sleep behavior disorder (RBD)^12^, etc. In addition to α-synuclein, other pathological proteins, such as β amyloid (Aβ) and tau pathology were increasingly reported to be associated with a part of non-motor symptoms of PD. It has been reported that the decreased Aβ level in CSF was related to memory impairment in PD patients with dementia (PDD)^13^. Aβ_1–42_ level in CSF in PD with fatigue group was lower than that in the non-fatigue group^10^. One study showed that de novo PD patients with PIGD phenotype had significantly reduced Aβ_1–42_ level in CSF compared with patients with TD phenotype and controls^14^. However, all of the studies recruited a “pure” population, either drug-naïve patients or demented patients, who were not quite appropriate for clinical distribution of PD patients. We previously reported that the levels of total tau (T-tau) and tau phosphorylated at the position of serine 396 (P-tau396s) in CSF from PD patients with mild cognitive impairment (MCI) were negatively correlated with MoCA score^15^. One study found increased T-tau level in CSF of PDD patients^16^. However, another study exhibited that decreased tau phosphorylated at the position of threonine 181(P-tau181t) level in CSF was significantly correlated with the postural instability in drug-naïve PD patients^17^. There were no determined answers regarding the role and mechanism of tau pathology in PIGD phenotype of PD due to the lack of large scale study. Based on the above analyses, we explored the role and mechanism of these pathological proteins in the cognitive impairment in PD patients with different motor phenotypes.

The decreased levels of dihydroxyphenylacetate (DOPAC) and homovanillic acid (HVA) in CSF, the two metabolites of dopamine, have been observed in PD patients as compared to controls^18^,^19^,^20^. Further study showed that PIGD phenotype responded poorly to dopaminergic treatment, whereas TD phenotype responded better^21^, indicating more involvement of dopaminergic networks in TD phenotype than in PIGD phenotype. One recent study showed that the elevated GABA level in plasma might be the biochemical basis of the PIGD phenotype of PD^22^. Cholinesterase inhibitors significantly reduced fall frequency in PD patients^23^. Stimulation of pedunculopontine nucleus (PPN) with low frequency improved gait abnormality and increased acetylcholine (Ach) level in the ventrolateral thalamic nucleus in 6-OHDA Parkinsonian rat^24^. Above studies indicated multiple neural pathways contributing to the PIGD phenotype of PD. However, there was no study investigating the potential neurochemical mechanisms underlying the correlation of different motor phenotypes with cognitive function in PD patients.

## Methods

### Subjects

#### Patients with PD

In total, 520 PD patients were recruited from the Department of Geriatrics and Neurology, Beijing Tiantan Hospital, Capital Medical University. Demographic information, including age, age of onset, sex, education, disease duration, predominantly affected side, disease severity, Montreal Cognitive Assessment (MoCA)-Beijing version^25^, Mini-Mental State Examination (MMSE) and levodopa equivalent daily dose (LED) were recorded. Patients were diagnosed with PD according to the criteria of Parkinson’s UK Brain Bank^26^.

#### Control subjects

In total, 28 age-matched controls from the Department of Geriatrics and Neurology, Beijing Tiantan Hospital were selected. The inclusion criteria were as follows: (1) no neurological symptoms and signs; (2) no intracranial diseases; (3) no essential tremor, PD, secondary parkinsonism, or Parkinson-plus syndrome; (4) no dysarthria or mental illness that affected expression; (5) no alcohol or drug abuse. All these controls had no family history of PD or tremor.

The controls were also patients, but their diseases, such as peripheral neuropathy and headache caused by high intracranial pressure, etc, were not related to and had no effect on the results of this investigation.

### Assessments of disease severity and motor symptoms of PD patients

The severity of PD was assessed by Hoehn and Yahr (H-Y) stage.

Each PD patient was evaluated using the items in the Unified Parkinson’s Disease Rating Scale (UPDRS) III, in which items 20 and 21 were for tremor, item 22 was for rigidity, items 23–26 and 31 were for bradykinesia, and items 27–30 were for PIGD. The score for each motor symptom was calculated by summing up the scores of corresponding items in UPDRS III.

Motor phenotypes were determined as either TD phenotype (n = 309) or PIGD (n = 211) phenotype following the classification algorithm proposed by Jankovic *et al*.^27^. Following the original classification methods, the ratio of the mean UPDRS tremor scores (8 items) to the mean UPDRS PIGD scores (5 items) was used to define TD phenotype (ratio ≥ 1.5), PIGD phenotype (ratio ≤ 1), or indeterminate phenotype (ratios >1.0 and <1.5). Finally, total 520 patients with PD were eligible for this study with 60 cases excluded due to the indeterminate motor phenotype.

### Assessment of cognitive function for PD patients

Cognitive function of PD patients was evaluated by the MoCA-Beijing version^25^ and MMSE. The MoCA-Beijing required education adjustment, i.e., one point was added to the total score for those with education <12 years^28^. The MoCA-Beijing had 7 cognitive domains, including visuospatial/executive function, attention, recall, orientation, abstract, language and naming. Patients with MoCA-Beijing score >26 points were defined as cognitively normal. We compared the score of each item and the percentage of completely correct in each item of MoCA-Beijing between PIGD group and TD group.

Beijing Tiantan Hospital review board (KY2013-003-03) has approved this study. All participants completed the written informed consents. This study met the guidelines of Capital Medical University, which abode by the Helsinki Declaration on ethical principles for medical research involving human subjects.

### Collections of CSF and serum samples

Before fasting, CSF (3 mL in a polypropylene tube via lumbar puncture) was collected from patients with PD whose conditions allowed us to withdraw the antiparkinsonian drugs for 12 to14 hours. Approximately 0.5 ml volume of CSF was aliquotted into separate Nunc cryotubes and kept frozen at −80 °C until used in assays. Each aliquot dedicated for each measure to avoid freeze-thawing and potential degradation of protein.

### Measurements of the levels of pathological proteins in CSF

The levels of pathological proteins, including Aβ_1–42_, T-tau, P-tau181t, P-tau396s and tau phosphorylated at the following positions: threonine 231 (P-tau231t) and serine 199 (P-tau199s), in CSF from PD patients and control participants were determined by using an enzyme-linked immunosorbent assay.

CSB-E10684h and CSBE12011h kits for measuring Aβ_1–42_ and T-tau, respectively, were obtained from CUSABIO (Wuhan, China). KHB7031, KHB7041, KHB8051 and KHO0631 kits for measuring P-tau396s, P-tau199s, P-tau231t and P-tau181t, respectively, were obtained from Invitrogen (Carlsbad, CA, USA).

### Measurements of the levels of neurotransmitters in CSF

The levels of DA and its two metabolites of HVA, DOPAC, Ach, serotonin (5-HT) and norepinephrine (NE) in CSF from PD patients were tested by high performance liquid chromatography (HPLC). Henomenex 150*2 mm, 150*3 mm chromatographic columns and LC-MS-MS 6410 chromatographic instrument were from Agilent Company (USA), and standard sample was from Sigma Company (USA).

### Data analyses

Statistical analyses were performed with SPSS Statistics 20.0 (IBM Corporation, New York, USA).

Demographic information, motor symptoms and MoCA score were compared between TD and PIGD groups. The levels of Aβ_1–42_, T-tau, P-tau181t, P-tau231t, P-tau396s and P-tau199s in CSF were compared among control, TD and PIGD groups.

Continuous variables, if they were normally distributed, were presented as means ± standard deviations and compared by ANOVA test. Bonferroni correction was performed in further comparisons between two groups. P value was significant when it was <0.017. Continuous variables, if they were not normally distributed, were presented as median (quartile) and compared by nonparametric test. P value was significant when it was <0.017 in further comparisons between two groups. Discrete variables were compared by Chi square test.

Spearman correlation analyses were made between PIGD score and the levels of Aβ_1–42_, T-tau, P-tau181t, P-tau231t, P-tau396s, and P-tau199s in CSF, between PIGD score and the levels of neurotransmitters in CSF, and between the levels of Aβ_1–42_ and P-tau181t and HVA level in CSF in PD group.

Multiple linear regression models were established, in which PIGD score in PD group was set as dependent variable, whereas age, age of onset, sex, education, predominantly affected side, MoCA score, disease duration, H-Y stage, Aβ_1–42_ and P-tau181t were set as independent variables. P value was significant when it was <0.05.

## Results

### Demographic and clinical variables of control, TD and PIGD groups

Among the 520 PD patients, 279 cases (53.63%) were male and 241 (46.37%) were female. The disease duration varied from 3 month to 30 years, with a median of 2.75 years [interquartile range (IQR): 4.0 years]. The average H-Y stage was 1.99 ± 0.80 stage. The demographic variables of PD patients were listed in Table 1.

PIGD group displayed significantly higher PIGD score (p < 0.001) and lower tremor score (p < 0.001) than the TD group, as well as higher scores of rigidity (p = 0.04) and bradykinesia (p < 0.001). PIGD group had significantly more patients at H-Y stage of 4–5 than TD group.

The percentage of completely correct in the item of recall in MoCA in PIGD group was significantly lower than that in TD group (p = 0.04). No statistical differences were seen in age, age of onset, gender, education, predominantly affected side and LED. There were no significant differences in total scores of MoCA, MMSE and the score of each item in MoCA, as well as the percentage of completely correct in other items between PIGD group and TD group (Table 1).

### The levels of pathological proteins in CSF from control, TD and PIGD groups

In TD and PIGD groups, Aβ_1–42_ level in CSF was prominently decreased and the levels of T-tau, P-tau181t, P-tau231t, P-tau396s and P-tau199s in CSF were all significantly elevated when compared with control group (Table 2).

In PIGD group, Aβ_1–42_ level in CSF was significantly reduced and P-tau181t level in CSF was evidently increased when compared with TD group (Table 2). P-tau199s level in CSF was elevated when compared with TD group (Table 2).

### The levels of neurotransmitters in CSF from control, TD and PIGD groups

In TD and PIGD groups, the levels of Ach, HVA and 5-HT in CSF were prominently lower than those in control subjects. Ach level in CSF in PIGD group was significantly lower than that in TD group (Table 2).

In PIGD group, the levels of HVA and 5-HT in CSF were decreased when compared with TD group (Table 2).

No significant differences in the levels of DA, DOPAC and NE in CSF were seen among the three groups (Table 2).

### Relationships between pathological proteins in CSF and motor phenotypes of PD

Univariate linear regression model showed that PIGD severity in PD patients was prominently and negatively correlated with MoCA score (β = −0.231, p = 0.000), education (β = −0.092, p = 0.030) and Aβ_1–42_ level in CSF (β = −0.221, p = 0.018), and positively correlated with age(β = 0.181, p = 0.000), disease duration (β = 0.300, p = 0.000), H-Y stage (β = 0.653, p = 0.000) and P-tau181t level in CSF (β = 0.224, p = 0.000). Further multivariate linear regression models with adjustment for potential confounders revealed significant associations of PIGD severity with reduced MoCA score, decreased Aβ_1–42_ level and increased P-tau181t level in CSF (Table 3).

Univariate linear regression model showed that tremor severity in PD patients was significantly correlated with older age (β = 0.180, p = 0.00), lower education level (β = −0.090, p = 0.030), lower MoCA score (β = −0.231, p = 0.000), longer disease duration (β = 0.300, p = 0.000), more advanced H-Y stage (β = 0.651, p = 0.000), lower Aβ_1–42_ level (β = −0.220, p = 0.018) and higher P-tau181t level (β = 0.220, p = 0.000) in CSF. Further multivariate linear regression models with adjustment for potential confounders demonstrated that H-Y stage was the only influencing factor for tremor (β = 1.466, p = 0.015) (Supplemental Table 1).

### Relationships between PIGD score and the levels of neurotransmitters in CSF from PIGD group

In total PD patients, there were no correlations between PIGD score and the levels of neurotransmitters in CSF. However, in PIGD group, PIGD score was prominently and negatively correlated with HVA level in CSF (r = −0.575, p = 0.016) (Table 4).

### Relationships between the levels of Aβ_1–42_ and neurotransmitters in CSF from PIGD group

In PIGD group, we furtherly conducted correlation between the levels of Aβ_1–42_, P-tau181t and HVA level in CSF. Correlation analyses showed that Aβ_1–42_ level was significantly and positively correlated with HVA level in CSF (r = 0.588, p = 0.035) (Supplemental Table 2).

## Discussion

In this study, PIGD group displayed significantly higher scores of PIGD, rigidity and bradykinesia than those in TD group (Table 1). Previous longitudinal studies reported that PIGD score worsened at similar rates as bradykinesia and rigidity, whereas tremor score was stable over time^29^,^30^. Thus, the development of symptoms and signs of PIGD might be one of the pivotal element of PD progression. This study indicated that PIGD group had more patients at H-Y stage 4–5 than those in TD group. Studies showed difference^31^ or no difference^32^ in the disease severity scored by the H-Y stage between PIGD group and TD group. As disease progressed, PD patients might gradually developed cognitive impairment, thus, disease severity revealed by more advanced H-Y stage might be related to cognitive deterioration.

In this study, the percentage of completely correct in the item of recall in MoCA in PIGD group was significantly lower than that in TD group (Supplemental Table 3), indicating that more PD patients with PIGD phenotype suffered from cognitive impairment. In the correlation analysis, PIGD score was significantly and negatively correlated with MoCA score (Table 3), which was consistent with the hypothesis that cognitive impairment might be associated with the PIGD subtype. Postural control is referred to the control of the body’s position with the aim of maintenance of balance and orientation^33^,^34^. A previous study showed that more cognition-related regions, such as caudate, fusiform gyrus and inferior parietal lobes^35^, were affected in PIGD patients. One study reported that cognitive performance was a predictor of postural control in individuals with PD^36^. PD patients could increase attention to counteract the balance disturbance in late stage^37^, therefore, PD patients exhibited more impaired postural control in the cognitive-motor dual-task condition in later stage because of distraction by cognitive task. Thus, cognitive-motor dual-task training could improve static postural control in PD patients^38^,^39^. These data implied that cognitive pathways might be involved in the PIGD phenotype.

We then investigated the potential mechanisms underlying the correlation between PIGD phenotype and cognitive impairment. Decreased Aβ_1–42_ level in CSF indicated Aβ aggregation and deposition in brain^9^. Substantial evidence suggested that lower Aβ_1–42_ level in CSF was related to cognitive impairment^40^. Previous study reported that Aβ_1–42_ level in CSF prominently decreased at later stage in PD patients with dementia^13^. In this study, our data demonstrated that PIGD severity was correlated with lower Aβ_1–42_ level in CSF, independent of age, education level, H-Y stage and cognition (Table 3). Thus, we speculated that PIGD might share common pathological mechanisms with cognitive impairment in PD patients featured by the increased Aβ_1–42_ aggregation in brain.

Under normal conditions, tau contributes to the integrity of the cytoskeleton. Excessive phosphorylation of tau in PD patients results in impaired cell integrity, loss of its physiological function and even cell death. Tau in CSF, thought to be a marker of neuronal death, was reported to be increased in PD patients with dementia^41^. Furthermore, increased tau level in CSF was found in PD patients with disease duration of less than 2 years, indicating that tau pathology might occur in the initial stage of the disease^42^. In this study, the results indicated that the levels of T-tau and all P-tau variables in PIGD group and TD group were significantly higher than those in control group. In total PD patients, PIGD severity was correlated with the increased P-tau181t level in CSF beyond age, education, H-Y stage and cognition (Table 3), implying that tau pathology might be a potential underlying mechanism of PIGD. Further comparison revealed that P-tau181t level in CSF in PIGD group was significantly higher than that in TD group (Table 2), indicating that PIGD patients might have severer or faster neurodegeneration than TD patients. P-tau181t was reported to be related to cognitive impairment in Alzheimer’s disease (AD) patients^43^, implying that P-tau181t might serve as a potential pathological marker linking PIGD and cognitive impairment in PD patients.

Up to now, there were just a few neurochemical studies, in which the levels of different neurotransmitters in CSF were examined in PD patients^44^. In this study, the data showed that HVA level in CSF in PIGD group and TD group were significantly lower than that in control group, and further comparison showed prominently declined HVA level in CSF in PIGD group compared with TD group (Table 3). We for the first time found that PIGD severity in PIGD group was significantly and negatively correlated with HVA level in CSF (Table 4), and a previous study reported that PIGD severity might be related to the degeneration of frontal dopaminergic pathways^45^, indicating that dopaminergic dysfunction was involved in PIGD. HVA was the main end-product of dopamine metabolism. Studies reported that the measurements of dopamine metabolites, rather than the parent compound, were the best reflection of dopamine’s moment-by-moment turnover in nerve terminals^30^,^46^.

Previous study reported that cholinergic dysfunction was related to gait disturbance in PD patients^47^. However, there was no study investigating the relationships between Ach level in CSF and motor phenotypes in PD. The present study was therefore the first to show that Ach level in CSF in PIGD group was remarkably decreased compared to that in TD group and control group (Table 2). This study demonstrated no association between Ach level in CSF and PIGD severity in PIGD group. Most of PIGD patients in our study were from out-patients clinic, and their PIGD symptoms were relatively mild. Therefore, the decreased Ach level in CSF might not play a key role in the neurobiological mechanisms of PIGD at that time.

Previous study demonstrated that aggravation of PIGD in advanced PD patients was related partially to impaired serotonergic transmission^48^. Reduced serotonergic neurotransmission impaired learning and memory function, whereas an increased serotonergic neurotransmission was associated with an improved cognitive and behavioral performance, not only in rodents, but also in AD patients^45^. A recent functional Magnetic Resonance Imaging (MRI) study demonstrated that the dysfunction of prefrontal-parietal network might be associated with the prominent gait impairments of PIGD subtype^35^. 5-HT projections originating from the raphe nuclei converged at several key target structures involved in memory, ascended within the medial forebrain bundle and were projected to prefrontal cortex^45^. Based on the above studies, it implied that the 5-HT projections abnormity might have an impact on PIGD. This study showed that 5-HT level in CSF in PIGD group and TD group were significantly lower than that in control group; furthermore, in PIGD group, 5-HT level in CSF in PIGD group was decreased when compared with TD group (Table 2). Yet, there was no correlation between PIGD severity and 5-HT level in CSF, which might be explained that patients in our study were at relatively early stage and the role of 5-HT dysfunction played on cognition in PIGD patients might not be significant.

This study also explored the relationship between the levels of neurotransmitters and Aβ_1–42_, T-tau and P-tau in CSF in PIGD group. Data showed that Aβ_1–42_ level in CSF was prominently and positively correlated with HVA level in CSF (Supplemental Table 2), indicating that Aβ_1–42_ level might accelerate dopamine degradation, causing the decreased HVA level in CSF and finally leading to PIGD.

In this investigation, P-tau_181t_ level in CSF was not correlated with each neurotransmitter level in CSF, which might be explained that most of PD patients were not demented (75.86% PD patients had MMSE >24 scores) and a mount of them (64.81%) were at the stage of no more than H-Y stage 2. We might, therefore, speculate that Aβ_1–42_ played a major role on cognitive impairment for PD patients at early stage, consistent with a recent study reporting that tau probably revealed the degree of neurodegeneration, whereas Aβ_1–42_ was a more specific marker for cognitive impairment.

In summary, the findings of this study revealed a relationship between PIGD and cognitive impairment in PD patients. The results here provided a neuropathological mechanism underlying the correlation between PIGD and cognitive impairment. The decreased HVA level in CSF was closely associated with PIGD severity in PD patients. Aβ_1–42_ deposition might contribute to HVA dysfunction in brain related to both PIGD and cognitive impairment in PD patients.

There are following limitations in this study. Relatively few CSF samples were analyzed due to the difficulties of obtaining CSF from PD patients and controls, which might weaken the statistical power of the analyses, such as of the correlations between PIGD severity with the levels of 5-HT and Ach in CSF. Cognitive function evaluated by MoCA was relatively rough and a formal neuropsychological test battery containing detailed cognitive domains would be used in the future study in order to provide more information.

## Additional Information

**How to cite this article**: Zuo, L.-J. *et al*. Phenotype of postural instability/gait difficulty in Parkinson disease: relevance to cognitive impairment and mechanism relating pathological proteins and neurotransmitters. *Sci. Rep.* **7**, 44872; doi: 10.1038/srep44872 (2017).

**Publisher's note:** Springer Nature remains neutral with regard to jurisdictional claims in published maps and institutional affiliations.

## Supplementary Material

Supplementary Information

## Figures and Tables

**Table 1:** Demographic information, motor symptoms and non-motor symptoms of TD and PIGD groups.

	Total PD patients	TD group	PIGD group	P value
	(520 cases)	(309 cases)	(211 cases)	
Age (years, mean ± SD)	61.37 ± 10.14	61.22 ± 0.38	61.73 ± 9.668	0.54
Age of onset (years, mean ± SD)	57.57 ± 11.03	57.66 ± 10.9	57.47 ± 10.92	0.84
Male/Total [cases/total (%)]	279/520(53.65)	158/309(51.13)	121/211(57.35)	0.80
Disease duration (years, mean ± SD)	3.91 ± 4.69	3.62 ± 3.79	4.46 ± 5.49	0.11
Education (N, %)				0.10
Primary school and below	154/520(29.62)	98/309(31.72)	56/211(26.54)	
Middle and high school	259/520(49.81)	154/309(49.84)	105/211(49.76)	
Bachelor’s degree and above	107/520(20.58)	57/309(18.45)	50/211(23.70)	
Hoehn-Yahr stage (N, %)				0.041*
Stage 0–1	108/520(20.77)	64/309(20.71)	44/211(20.85)	0.527
Stage 2	229/520(44.04)	148/309(47.90)	81/211(38.39)	0.038*
Stage 3	165/520(31.73)	94/309(30.42)	71/211(33.65)	0.248
Stage 4–5	18/520(3.46)	3/309(0.97)	15/211(7.11)	0.000**
UPDRS III (years, mean ± SD)	25.97 ± 13.83	25.50 ± 12.93	27.49 ± 14.5	0.12
Tremor	5.48 ± 3.74	6.67 ± 3.76	3.66 ± 2.56	0.00**
Rigidity	5.73 ± 3.76	5.42 ± 3.64	6.08 ± 3.88	0.04*
Bradykinesia	10.75 ± 6.55	10.05 ± 5.89	11.91 ± 7.11	0.00**
Postural and gait abnormalities	4.18 ± 2.73	3.45 ± 2.05	5.22 ± 3.06	0.00**
Predominantly affected side (N, %)				0.11
Left side predominantly affected	237/520(45.58)	142/309(45.95)	95/211(45.02)	
Right side predominantly affected	283/520(54.42)	167/309(54.05)	116/211(54.98)	
MoCA (scores, mean ± SD)	21.35 ± 6.11	21.51 ± 6.39	21.02 ± 6.59	0.28
Levodopa equivalent dose (LED) (mg, mean ± SD)	321.76 ± 109.23	319.56 ± 109.67	324.13 ± 104.29	0.32

TD = tremor-dominant; PIGD = postural instability/gait difficulty; UPDRS = Unified Parkinson’s Disease Rating Scale; MoCA = Montreal Cognitive Assessment; P: TD group vs. PIGD group. *P < 0.05, **P < 0.01.

**Table 2:** The levels of pathological proteins and neurotransmitters in CSF from control, TD and PIGD groups.

	Control group	TD group	PIGD group	P1 value	P2 value	P3 value
	(n = 28 cases)	(n = 78 cases)	(n = 44 cases)			
Aβ_1–42_ (ng/ml, mean ± SD)	0.87 ± 0.44	0.67 ± 0.33	0.52 ± 0.36	0.00**	0.00*	0.013*
T-tau [(pg/ml, (median (quartile)]	46.75 (10.27~158.06)	100.15 (51.27~142.31)	122.81 (55.52~177.94)	0.00**	0.00**	0.41
P-tau231t (pg/ml, mean ± SD)	65.59 ± 23.66	164.03 ± 76.33	148.91 ± 77.22	0.00**	0.00**	0.35
P-tau181t (pg/ml, mean ± SD)	42.10 ± 12.34	67.94 ± 27.40	90.49 ± 30.78	0.00**	0.00**	0.00**
P-tau199s (pg/ml, mean ± SD)	3.41 ± 0.57	7.23 ± 3.07	8.55 ± 3.78	0.00**	0.00**	0.06
P-tau396s (pg/ml, mean ± SD)	50.4 ± 26.17	67.41 ± 28.23	71.43 ± 33.54	0.01*	0.01*	0.54
Neurotransmitters (ng/ml*10^−2^, mean ± SD)						
Ach	0.70 ± 0.31	0.51 ± 0.13	0.30 ± 0.26	0.008*	0.00**	0.000*
DA	0.67 ± 0.30	0.66 ± 0.35	0.65 ± 0.25	0.89	0.87	0.92
HVA	0.15 ± 0.05	0.06 ± 0.03	0.04 ± 0.02	0.00**	0.00**	0.09
DOPAC	0.16 ± 0.07	0.16 ± 0.06	0.15 ± 0.06	0.60	0.31	0.29
5-HT	2.10 ± 0.82	1.50 ± 0.90	1.39 ± 0.73	0.00**	0.00**	0.07
NE	55.65 ± 15.73	1.52 ± 4.23	49.40 ± 13.63	0.35	0.13	0.48

Kruskal-Wallis test was used to compare T-tau level in CSF among control, TD and PIGD groups; ANOVA was used to compare the levels of Aβ_1–42_, P-tau231t, P-tau181t, P-tau199s, P-tau396s, Ach, DA, HVA, DOPAC, 5-HT and NE in CSF among control, TD and PIGD groups; *p* < 0.017 was defined as statistically significant.

P1: Control group vs. TD group; Kruskal-Wallis test was used to compare T-tau level in CSF between control and TD groups; *p* < 0.017 was defined as statistically significant. Two-tailed t-test was used to compare the levels of Aβ_1–42_, P-tau231t, P-tau181t, P-tau199s, P-tau396s, Ach, DA, HVA, DOPAC, 5-HT and NE in CSF between control and TD groups, *p* < 0.017 was defined as statistically significant. P2: Control group vs. PIGD group; Kruskal-Wallis test was used to compare T-tau level in CSF between control and PIGD groups; *p* < 0.017 was defined as statistically significant. Two-tailed t-test was used to compare the levels of Aβ_1–42_, P-tau231t, P-tau181t, P-tau199s, P-tau396s, Ach, DA, HVA, DOPAC, 5-HT and NE in CSF between control and PIGD groups; *p* < 0.0017 was defined as statistically significant.

P3: TD group vs. PIGD group; Kruskal-Wallis test was used to compare T-tau level in CSF between TD and PIGD groups; *p* < 0.017 was defined as statistically significant. Two-tailed t-test was used to compare CSF levels of Aβ_1–42_, P-tau231t, P-tau181t, P-tau199s, P-tau396s, Ach, DA, HVA, DOPAC, 5-HT and NE between TD and PIGD groups; *p* < 0.017 was defined as statistically significant. Aβ_1–42_ = β amyloid (Aβ)_1–42_; T-tau = total tau; P-tau181t = tau phosphorylated at threonine 181; P-tau231t = tau phosphorylated at threonine231; P-tau396s = tau phosphorylated at serine 396; P-tau 199s = tau phosphorylated at serine 199; Ach = acetylcholine; DA = dopamine; DOPAC = dihydroxyphenylacetate; HVA = homovanillic acid; 5-HT = serotonin; NE = norepinephrine. *P < 0.017, **P < 0.01.

**Table 3:** Associations of PIGD severity with the levels of Aβ_1–42_ and P-tau181t in CSF in univariate and multivariate linear regression models with adjustment for potential confounders.

Variable	Univariate		Multivariate			
	β	P value	β	P value	β	P value
Age (years)	0.181	0.000**	0.009	0.876	0.640	0.893
Age of onset (years)	0.049	0.253	−0.032	0.923	−0.020	0.803
Sex (male/total, %)	−0.007	0.859	−0.288	0.422	0.057	0.883
Education (N, %)	−0.092	0.030*	0.022	0.945	0.342	0.311
Predominantly affected side	0.008	0.861	−0.240	0.395	0.018	0.73
MoCA (scores)	−0.231	0.000**	−0.066	0.048*	0.037	0.047*
Disease duration (years)	0.300	0.000**	0.126	0.288	0.059	0.624
Hoehn-Yahr stage (stage)	0.653	0.000**	2.130	0.000**	2.247	0.000**
Aβ_1–42_ in CSF (ng/ml)	−0.221	0.018*	−1.178	0.014*	—	—
P-tau181t in CSF (pg/ml)	0.224	0.000**	—	—	0.018	0.012*

MoCA = Montreal Cognitive Assessment; Aβ_1–42  _ = β amyloid (Aβ)_1–42_; P-tau181t = tau phosphorylated at threonine 181. *P < 0.05, **P < 0.01.

**Table 4:** Correlation between PIGD severity and HVA level in CSF from PIGD group.

PIGD severity (points)	R	P value
HVA in CSF (ng/mL)	−0.575	0.016*

HVA = homovanillic acid. *P < 0.05.
