# Supplementary material for: Phenotype of postural instability/gait difficulty in Parkinson disease: relevance to cognitive impairment and mechanism relating pathological proteins and neurotransmitters
Source: Sci Rep. 2017 Mar 23;7:44872. doi: 10.1038/srep44872 (PMC5362957; doi:10.1038/srep44872)
Supplement: Supplementary Information [file srep44872-s1.pdf]

**Phenotype of postural instability/gait difficulty in Parkinson disease:**

**relevance to cognitive impairment and mechanism relating pathological proteins and neurotransmitters**

Li-Jun ZUO MD[1], Ying-Shan PIAO MD, PhD[2], Li-Xia LI MD[2], Shu-Yang YU MD[2], Peng GUO MD[2], Yang HU MD[2], Teng-Hong LIAN MD[1], Rui-Dan WANG MD[1], Qiu-Jin YU MD[1], Zhao JIN MD[1], Ya-Jie WANG PhD[3], Xiao-Min WANG PhD[4,5,6,7], Piu CHAN MD, PhD[8,5,7,6], Sheng-Di CHEN MD, PhD[9], Yong-Jun WANG MD[1,10], Wei ZHANG MD, PhD[2,1,10,5,6,7]

**Supplemental table 1 Associations of tremor severity with the levels of $A\beta_{1-42}$ and P-tau181t in CSF in univariate and multivariate linear regression models with adjustment for potential confounder**

| | Univariate | | Multivariate | | | |
|---|---|---|---|---|---|---|
| Variable | β | P value | β | P value | β | P value |
| Age (years) | 0.180 | **0.00**** | 0.031 | 0.883 | 0.043 | 0.845 |
| Age of onset(years) | 0.049 | 0.253 | -0.053 | 0.794 | -0.051 | 0.812 |
| sex (male/total, %) | -0.007 | 0.859 | -0.076 | 0.922 | 0.232 | 0.800 |
| Education (N, %) | -0.090 | **0.030*** | -0.167 | 0.813 | -0.489 | 0.214 |
| Predominantly affected side | 0.008 | 0.862 | 0.496 | 0.426 | 0.984 | 0.198 |
| MoCA (scores) | -0.231 | **0.00**** | -0.090 | 0.254 | -0.016 | 0.862 |
| Disease duration (years) | 0.300 | **0.00**** | 0.246 | 0.345 | 0.354 | 0.985 |
| Hoehn-Yahr stage (N, %) | 0.651 | **0.00**** | 1.534 | **0.003**** | 1.466 | **0.015*** |
| $A\beta_{1-42}$ in CSF(ng/ml) | -0.220 | **0.018*** | 1.271 | 0.222 | ---- | ---- |
| P-tau181t in CSF (pg/ml) | 0.220 | **0.00**** | ---- | ---- | 0.022 | 0.192 |

MoCA= Montreal Cognitive Assessment; $A\beta_{1-42}$ =β amyloid $(A\beta)_{1-42}$; P-tau181t= tau phosphorylated at threonine 181.*P＜0.05,**P＜0.01.

**Supplemental table 2 Correlation between the levels of HVA and $A\beta_{1-42}$ in CSF from PIGD group**

| $A\beta_{1-42}$ in CSF (ng/ml) | R | P value |
|---|---|---|
| HVA in CSF (ng/mL) | 0.588 | 0.035* |

HVA= homovanillic acid. *P＜0.05.

**Supplemental table 3 Comparison of percentage of MoCA subtest full scores between PIGD group and TD group**

| | Total PD patients ( 520 cases) | TD group ( 309 cases) | PIGD group ( 211 cases) | P value |
|---|---|---|---|---|
| Visuospatial/Executive function(5) | 113/520(21.73%) | 72/309(23.30%) | 41/211(19.43%) | 0.17 |
| Attention(6) | 274/520(52.69%) | 161/309(52.10%) | 113/211(53.55%) | 0.41 |
| Recall(5) | 43/520(8.27%) | 31/309(10.03%) | 12/211 (5.69%) | **0.04*** |
| Orientation(6) | 273/520(52.50%) | 220/309(71.20%) | 153/211(72.51%) | 0.41 |
| Abstraction(2) | 215/520(41.35%) | 123/309(39.81%) | 92/211(43.60%) | 0.22 |
| Language(3) | 227/520(43.65%) | 141/309(45.63%) | 86/211(40.76%) | 0.156 |
| Naming(3) | 375/520(72.12%) | 224/309(72.49%) | 151/211(71.56%) | 0.45 |

P: TD group vs. PIGD group. *P＜0.05.

**Supplemental table 4 Comparison of percentage of major depression between TD and PIGD groups**

| | Total PD patients ( 520 cases) | TD group ( 309 cases) | PIGD group ( 211 cases) | P value |
|---|---|---|---|---|

| | | | | |
|---|---|---|---|---|
| HAMD＞34 (N, %) | 96/520(18.46%) | 65/309(21.04%) | 31/211(14.69%) | **0.04*** |

HAMD = Hamilton Depression Scale (24 items).*P＜0.05.
